# Historic Mining and Agriculture as Indicators of Occurrence and Abundance of Widespread Invasive Plant Species

**DOI:** 10.1371/journal.pone.0128161

**Published:** 2015-06-05

**Authors:** Kellen Calinger, Elisabeth Calhoon, Hsiao-chi Chang, James Whitacre, John Wenzel, Liza Comita, Simon Queenborough

**Affiliations:** 1 Department of Evolution, Ecology, and Organismal Biology, The Ohio State University, 318 W. 12th Avenue, Columbus, Ohio 43210–1293, United States of America; 2 Powdermill Nature Reserve, Carnegie Museum of Natural History, 1847 Route 381, Rector, Pennsylvania 15677, United States of America; 3 Smithsonian Tropical Research Institute, Box 0843–03092, Balboa, Ancón, Panama; 4 University Library, University of Illinois, Urbana, Illinois 61801, United States of America; Shandong University, CHINA

## Abstract

Anthropogenic disturbances often change ecological communities and provide opportunities for non-native species invasion. Understanding the impacts of disturbances on species invasion is therefore crucial for invasive species management. We used generalized linear mixed effects models to explore the influence of land-use history and distance to roads on the occurrence and abundance of two invasive plant species (*Rosa multiflora* and *Berberis thunbergii*) in a 900-ha deciduous forest in the eastern U.S.A., the Powdermill Nature Reserve. Although much of the reserve has been continuously forested since at least 1939, aerial photos revealed a variety of land-uses since then including agriculture, mining, logging, and development. By 2008, both *R*. *multiflora* and *B*. *thunbergii* were widespread throughout the reserve (occurring in 24% and 13% of 4417 10-m diameter regularly-placed vegetation plots, respectively) with occurrence and abundance of each varying significantly with land-use history. *Rosa multiflora* was more likely to occur in historically farmed, mined, logged or developed plots than in plots that remained forested, (log odds of 1.8 to 3.0); *Berberis thunbergii* was more likely to occur in plots with agricultural, mining, or logging history than in plots without disturbance (log odds of 1.4 to 2.1). Mining, logging, and agriculture increased the probability that *R*. *multiflora* had >10% cover while only past agriculture was related to cover of *B*. *thunbergii*. Proximity to roads was positively correlated with the occurrence of *R*. *multiflora* (a 0.26 increase in the log odds for every 1-m closer) but not *B*. *thunbergii*, and roads had no impact on the abundance of either species. Our results indicated that a wide variety of disturbances may aid the introduction of invasive species into new habitats, while high-impact disturbances such as agriculture and mining increase the likelihood of high abundance post-introduction.

## Introduction

A variety of factors determine whether a biological community is particularly susceptible to invasion, including soil nutrients, light levels, disturbance regime, and land-use history [1]. However, some questions remain as to how these factors interact and, in particular, how both the ability of a species to invade and its population abundance once it establishes are influenced by land-use history. The effects of some types of land use on invasive species establishment and spread have been studied in greater detail than others and most studies do not include the effects of distance to road or consider the combined effects of past and current land use.

Several studies have implicated past land use as a major factor in the establishment and spread of invasive plant species [1][2][3][4]. Changes in land use, such as clearing land for agriculture and then subsequent reforestation, often create “windows of opportunity” for invasive species to establish in these disturbed areas [1]. Agriculture, logging, and residential or commercial development all typically increase the distribution of invasive plants, although this depends on the species in question and land use seems to have more of an effect on occurrence than on abundance [1][2][3][5]. Additionally, past land use is more predictive of invasive occurrence and abundance than present land use [1][2], but the best predictor of distribution may be found by combining past and present land use into a single metric of land-use change [5], which is rarely done.

Furthermore, the effects of some past land uses are less well studied, despite their high prevalence in the landscape. For example, the effects of agriculture and logging on invasive spread have been extensively studied, whereas the effects of mining have not been studied as extensively or in the same ways. Over 400,000 hectares of forest have been lost to mining since 1973 in the eastern United States alone and coal mining is currently the leading cause of forest loss in the Allegheny Plateau ecoregion that contains our study site, Powdermill Nature Reserve [6]. Given that mining destroys vegetation, disrupts soils, and alters microbial communities [7], it seems likely that mining would enhance the spread of invasive organisms by also creating “windows of opportunity” in a similar manner to how agriculture enhances the spread of invasive species. This situation appears likely, but studies of mining impacts have generally focused on the prevalence of invasive species over the course of succession or on the effects of planting invasive species as part of the mine reclamation process on subsequent invasive species prevalence [8][9][10,11]. Previous studies have not compared the occurrence and spread of invasive species in historically mined areas to those that were historically forest or compared the effects of mining to the effects of other land uses.

Roads may also aid the spread of invasive species, even in areas that have historically been forested [12][13]. Roads provide suitable habitats for invasive species as well as corridors for the dispersal of invasive species [12][14]. However, only a few studies have combined models of invasive species presence and distance to roads with land-use history to examine whether roads continue to be important predictors of invasive spread when land use is included in the model and *vice versa* [2][15][16]. It is important to include both land use and distance to roads in models because the land use history and roads could potentially have interacting effects. For instance, a road close to a disturbed site could increase propagule pressure in that site [4]. The handful of studies that have made this comparison have found inconsistent results [2][15][16], potentially due to species-specific differences in the effects of roads on the occurrence and spread of invasive species [17].

In this exploratory study, we examined patterns of occurrence and abundance of two invasive plant species in a deciduous forest ecosystem in eastern North America as a function of roughly 70 years of land-use history and of distance to roads to ask the following questions: (1) How do patterns of land-use change including surface mining for coal, logging, agriculture, and development affect the occurrence and abundance of invasive species? (2) How does the distance to a road affect the occurrence and abundance of invasive species? (3) Is land-use change still a significant predictor of invasive occurrence and abundance once distance to road is accounted for and *vice versa*?

Multiflora rose (*Rosa multiflora*) and Japanese barberry (*Berberis thunbergii*) were the only invasive species in our study site that were present in large enough numbers to examine their distribution patterns individually. We predicted that historical land use change would have greater effects on occurrence of invasive species than abundance for both species in accordance with previous research [1][2][3][5]. We also expected that mining would have equal, or greater effects, than agriculture on the occurrence and abundance of these species, given that the effects of mining on the environment tend to be more long-lasting and severe than the effects of agriculture [11]. Lastly, we expected to observe species-specific differences in the effect of roads on invasive species occurrence and abundance, potentially with distance to road having greater effects on *B*. *thunbergii* than on *R*. *multiflora*, as was seen in previous research, although that study did not include the effects of land-use change in their model [13].

## Materials and Methods

### Study Site

To examine the impacts of land-use history on invasive species, we analyzed an extensive vegetation survey from Powdermill Nature Reserve (PNR; 40°09’S, 79°16’W), currently private property maintained as a research field station by the Carnegie Museum of Natural History in Pittsburgh, PA. PNR covers nearly 900 hectares of temperate deciduous forest in southwestern Pennsylvania (U.S.A), where the predominant tree taxa are maple (*Acer*), oak (*Quercus*), hickory (*Carya*), and tulip poplar (*Liriodendron*, [18] for more information on native species in PNR).

Although PNR is currently predominantly forested, the region has experienced a variety of anthropogenic disturbances. As with most forests in the eastern U.S., much of the reserve was logged in the 1800s. The majority of the reserve returned to forest afterward although a small fraction of the reserve experienced more recent clear cutting in the 1960s and became forested later. Following logging, parts of the property were used for agriculture until the early to mid-1900s. In addition, surface mining for coal occurred during the 1940s. Little development (e.g., building) has taken place since PNR was designated as a nature reserve and became a part of Carnegie Museum of Natural History in 1956. At present, most of the PNR remains forested and undisturbed with its east boundary connected to the 200-ha Laurel Mountain State Park. However, a high level of anthropogenic disturbance (e.g., residential areas and highways) has occurred to the west side of the reserve, which could have accelerated the introduction and establishment of invasive species. No special permission was required to measure woody plants on private property and no protected species were involved in the survey. This study was approved by the Director of Powdermill Nature Reserve and by the Director of the Carnegie Museum of Natural History.

### Data Collection

During the summers of 2006, 2007, and 2008, a continuous 120m x 120m grid was mapped across PNR (for a total of 647 'blocks'). Within each block, nine circular vegetation plots (10m in radius, ~314 m^2^ in area, total area = 2827 m^2^) were established in a square around the center of each block. Within each vegetation plot, all trees, shrubs, and herbs were surveyed. Shrub and herb abundance was assessed as percent cover (“cover class” hereafter) and assigned a numeric value between 0 and 6 (0, trace = 1, 1–10% = 2, 11–25% = 3, 26–50% = 4, 51–75% = 5, and >75% = 6). Based on cover class assignments, *R*. *multiflora* and *B*. *thunbergii* cover in each plot was binned into a “low cover” category (trace-10% cover, hereafter “low abundance”) and a “high cover” category (>10% cover, hereafter “high abundance).

**Fig 1:**
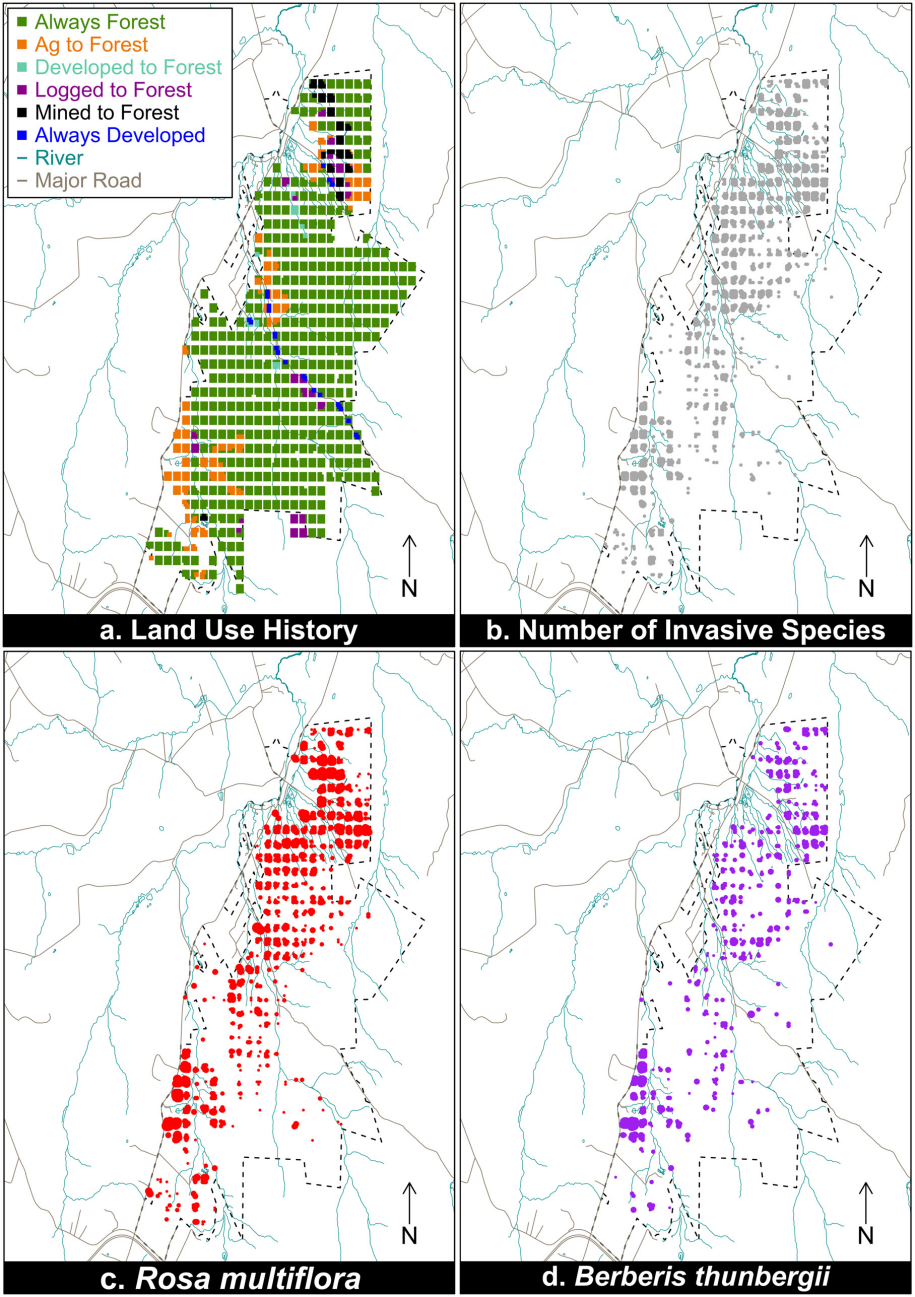
Land-use history (a) and abundance of invasive species in Powdermill Nature Reserve in 2006. Point sizes correspond to the number of invasive species present ranging from 1 to 3 invasive species present (b) and abundance of *R*. *multiflora* (c) and *B*. *thunbergii* (d). Blue lines represent rivers, while brown lines represent major roads in the region.

To document the various anthropogenic disturbances that have occurred at PNR, we used historical aerial images to assign land-use history categories to each vegetation plot. Aerial images were available from 1939, 1957, 1967, 1993, and 2006 [19]. We classified types of land-use history using the standard national land cover database classification system [20], which includes forest land, shrub land, planted/cultivated, and barren (including surface mining). Historical mining activities inside the property were matched with the aerial imaging. We ultimately classified each plot into one of the following six categories based on previous anthropogenic disturbances and current conditions (as suggested by Mosher et al. 2009 [5]): always forested (since at least 1939), agriculture to forest, mined to forest (all mining was surface mining for coal), logged to forest, developed to forest, and always developed (Fig 1a). Many of these land-use history categories represent patterns of secondary succession common throughout the eastern U.S.A.

Current human disturbance (e.g., buildings, yards, and trails) within a 10m radius of each plot was noted. Among the plots with a history of development, ~58% of them were open space with minimal construction materials (e.g., lawn grass) that later became forested. The rest of the plots had impervious surfaces accounting for 50–79% of the total cover (e.g., buildings). Distances from each plot to roads and forest edge were calculated in the ArcGIS software program [21].

### Study species

Five invasive plant species were identified in the survey plots at PNR. Multiflora rose (*Rosa multiflora*) and Japanese barberry (*Berberis thunbergii*) were widespread in the reserve (accounting for 65% and 34% of all invasive species occurrences, respectively), while the three remaining species combined accounted for <1.5% of invasive species coverage (*Lonicera morrowi*, *Alliaria petiolata*, and *Celastrus orbiculatus*, Fig 1). Only *R*. *multiflora* and *B*. *thunbergii* had sufficient sample sizes to be analyzed individually. All five invasive species were included for a third analysis assessing impacts of land-use history on the general occurrence of invasive species within PNR.

*Rosa multiflora* Thunb. is a thorny shrub, originally introduced to the U.S.A. as rootstock in 1866 from Eastern Asia [22][23] and it was later planted for erosion control and as fences and highway strips. A prolific species, a mature *R*. *multiflora* individual could produce up to one million seeds per year [24] and the fruits could remain viable for 20 years [25]. Besides seed dispersal by birds, vegetative spread is also common. *Rosa multiflora* is tolerant to various soils, moisture and light conditions, and can be found in open woodlands, forest edges and areas that have experienced disturbance. *Rosa multiflora* was introduced to Pennsylvania as rootstock during the 1930s through the 1960s [26].

*Berberis thunbergii* DC. is a dense spiny shrub that was introduced from Japan as an ornamental plant in 1875 [27], but it did not become feral in the Northeast U.S.A. until the 1910s [28]. *Berberis thunbergii* seeds are dispersed by birds and mammals, although expansion through vegetative growth is also common. *Berberis thunbergii* can tolerate both shade and drought; therefore, it could adapt to various environmental conditions, including wooded habitats, wetland and disturbed areas [28].

### Statistical Analyses

We evaluated the effects of land-use history (a six-level categorical variable) and distance to road (continuous, meters) on the occurrence and abundance of the two most abundant invasive plant species at PNR, *R*. *multiflora* and *B*. *thunbergii*, using generalized linear mixed effects models from the lme4 package [29] in the statistical software R [30].

First, to predict the occurrence of invasive plants, we ran a separate logistic regression for each species (one for *R*. *multiflora*, one for *B*. *thunbergii*) to model occurrence (presence/absence) as a function of land-use and distance to roads. Second, to predict the abundance of invasive plants (i.e., abundance in those sites that contained the invasive species, ignoring sites where they did not occur), we also ran two separate logistic regressions (one for *R*. *multiflora*, one for *B*. *thunbergii*) to model abundance as a function of land-use and distance to roads. In this case, we divided invasive plant abundance into two classes, low and high, based on percent cover assigned in the field, as stated above. Low abundance sites had trace-10% cover of the invasive plant; high abundance sites had >10% cover. All models of invasive species occurrence and abundance used a binomial error structure and a logit link function. Finally, to predict the number of invasive plant species, we ran a generalized linear model with a Poisson error structure, to model the number of invasive plant species as a function of land-use history and distance to roads. All models used a Type III sum of square errors.

For all models, we assigned the least disturbed “always forested” land-use category as the reference group against which all other land-use categories were compared. Distance to road (meters) was log transformed prior to analyses to normalize the data.

To account for potential spatial autocorrelation (or similarity between plots due to their proximity to each other) in invasive plant occurrence and abundance because of the vegetation sampling methods (blocks of nine 10m radius plots centered in each cell of a 120x120 m grid (see Materials and Methods/Data Collection), all models included block as a random effect. Plotting the model residuals indicated that the addition of block as a random effect removed the majority of the spatial autocorrelation from our models (Figures A–D in S1 Fig). There was no significant spatial autocorrelation in model residuals of occurrence or abundance for *B*. *thunbergii* after the additional of block as a random effect (Moran’s I, *p* > 0.05, Figures C–D in S1 Fig) and thus our results for these models are independent of spatial bias. Addition of block to the occurrence and abundance models for *R*. *multiflora* accounted for spatial autocorrelation in plots ≥50 meters apart (Figures A–B in S1 Fig). Plots that are closer than 50 meters to each other are within the same 120x120m block, so it is reasonable that addition of block as a random effect would not account for all spatial autocorrelation at smaller scales (Moran’s I, *p* < 0.05, Figures A–B in S1 Fig). The spatial autocorrelation within a block would primarily be of concern if *R*. *multiflora* was found in only one block for a given land-use category as that may suggest that *R*. *multiflora* was found more often due to some site characteristic of that block rather than its land-use history. However, *R*. *multiflora* was found in multiple blocks for all land-use categories assessed (S1 Table) suggesting that the observed differences in occurrence and abundance are not due to the remaining spatial autocorrelation in our models. Further, impacts of spatial autocorrelation are primarily of concern in studies with small sample sizes [27]. Our samples sizes for both *R*. *multiflora* and *B*. *thunbergii* are comparable to or larger than similar studies which had sample sizes ranging from n = 28 to n = 188 [1][2][11][14][31]. Given our large sample sizes for both *R*. *multiflora* and *B*. *thunbergii*, the remaining spatial autocorrelation in our model residuals is unlikely to significantly impact our results.

## Results

### Invasive species occurrence

**Fig 2:**
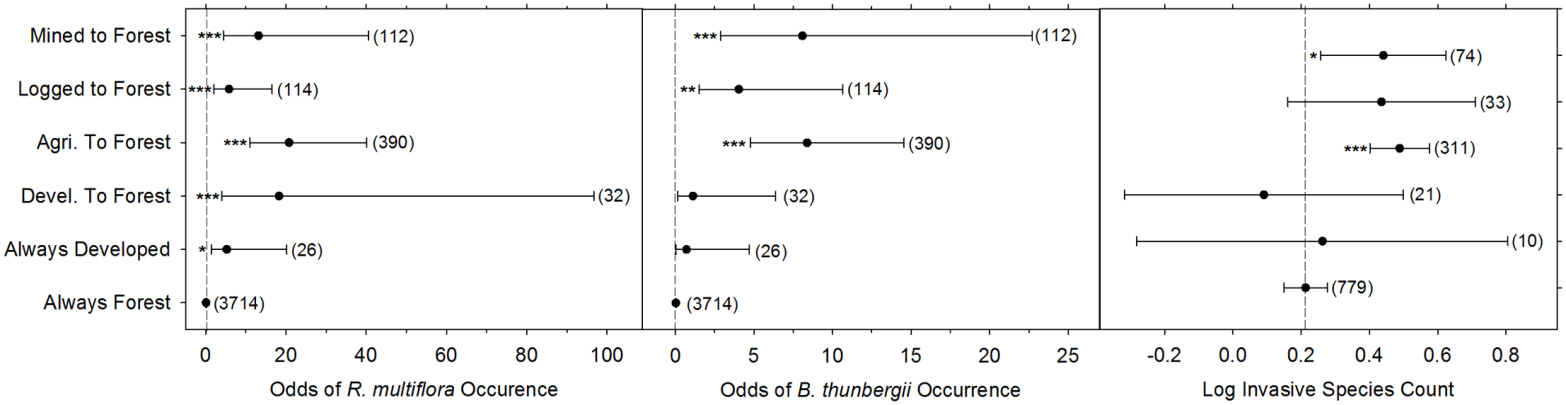
Plot-level odds of (a) *R*. *multiflora* and (b) *B*. *thunbergii* occurrence and (c) plot-level log odds of finding multiple invasive species. A total of 4388 10-m diameter plots with different land-use histories in a deciduous forest in eastern North America were included in this analysis except in the invasive species count analysis in which only plots with at least one invasive species were included (n = 792). The odds of occurrence for each invasive species in a given land-use category relative to always forested plots is given by each point with 95% confidence intervals. Sample sizes are given in parentheses. Among land-use histories, always forested plots (i.e. forested continuously since at least 1939) was used as the reference group and asterisks indicate a significant difference from always forested plots (*p < 0.05, **p < 0.01, ***p < 0.001).

*Rosa multiflora* was widespread throughout the reserve, occurring in 1080 plots (24%) and 258 blocks (50%, S1 Table). Occurrence varied by land-use history within PNR, with *R*. *multiflora* occurring in 18% of always forested plots, 74% of agriculture to forest plots, 65% of developed to forest plots, 27% of logged plots, 64% of mined plots, and 35% of developed plots (Fig 1c). As predicted, disturbance by humans increased the probability of invasion and the inclusion of land-use history significantly improved model fit (LR Χ^2^ = 111.4, df = 5, p < 0.001); the probability of occurrence of *R*. *multiflora* was significantly higher in all land-use categories compared to always forested sites (Fig 2). For example, for a plot that was previously mined the log odds of containing *R*. *multiflora* increased by 2.57 (Wald Z = 4.562, p < 0.001) and the odds by 13.16. For forest that had previously been logged, the log odds of containing *R*. *multiflora* increased by 1.76 (Wald Z = 3.348, p < 0.001) and the odds by 5.82. Previous agriculture was associated with the highest probability of invasion, with the log odds increasing by 3.03 (Wald Z = 9.214, p < 0.001) and the odds increasing by 20.78.

**Fig 3:**
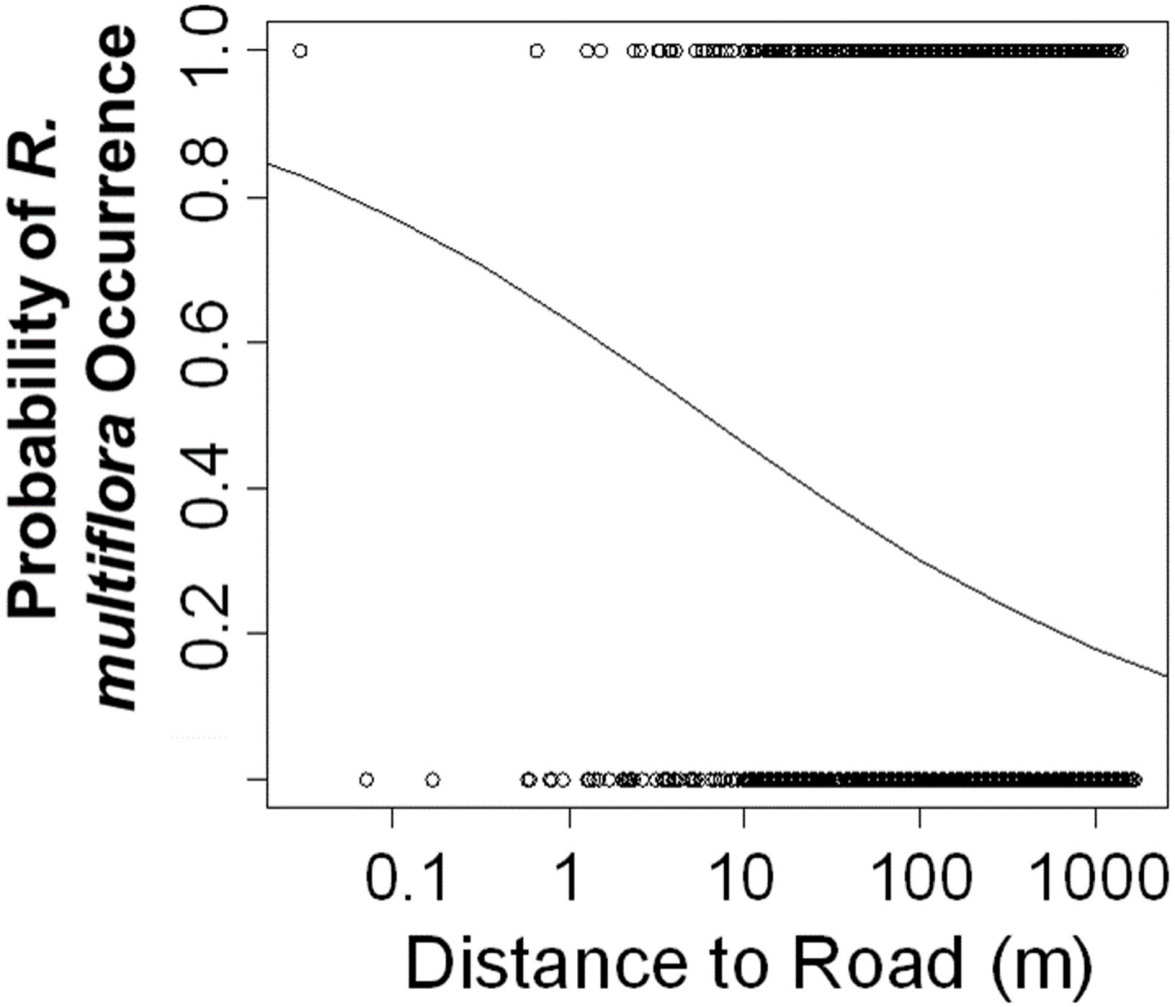
Probability of occurrence of *R*. *multiflora* versus distance to a road. Individual points represent plot-level observations of *R*. *multiflora*’s occurrence (1 = present, 0 = not present,n = 4388 plots). The best fit line was fitted using the generalized linear model of occurrence versus log distance to road and provides the probability of *R*. *multiflora* occurrence at varying distances from roadways.

As predicted, proximity to roads was positively correlated with the probability of invasion by *R*. *multiflora*, and inclusion of distance to road significantly improved model fit (LR Χ^2^ = 4.3676, df = 1, p = 0.037; Fig 3). For every 1-m closer to a road, the log odds that a plot contained *R*. *multiflora* increased by 0.26 (Wald Z = -2.14, p = 0.035, Fig 3).

*Berberis thunbergii* was rarer than *R*. *multiflora*, encountered in 563 plots (13%) and 190 blocks (37%) across the entire reserve (S1 Table). Occurrence varied by land-use history within PNR, and *B*. *thunbergii* occurred in 8% of always forested plots, 49% of agriculture to forest plots, 6% of developed to forest plots, 17% of logged plots, 36% of mined plots, and 4% of developed plots (Fig 1d). As predicted, disturbance by humans increased the probability of invasion and the inclusion of land-use history significantly improved model fit (LR Χ^2^ = 56.78, df = 5, p < 0.001); the probability of occurrence of *B*. *thunbergii* was significantly higher in all but two (developed to forest and always developed) land-use categories compared to always forested sites (Fig 2). For example, for a plot that was previously mined the log odds of containing *B*. *thunbergii* increased by 2.08 (Wald Z = 3.986, p < 0.001) and the odds by 8.08. For forest that had previously been logged, the log odds of containing *B*. *thunbergii* increased by 1.39 (Wald Z = 2.827, p = 0.0047) and the odds by 4.03. As with *R*. *multiflora*, previous agriculture was associated with the highest probability of invasion, with the log odds increasing by 2.13 (Wald Z = 7.509, p < 0.001) and the odds increasing by 8.38.

Unlike with *R*. *multiflora*, increasing proximity to roads did not significantly increase the probability of *B*. *thunbergii* occurrence (Wald Z = -1.856, *p* = 0.06, Fig 3b), and inclusion of distance to road did not significantly improve model fit (LR Χ^2^ = 3.34, df = 1, p = 0.067).

The total number of invasive species found in individual plots varied from 0 to 3. A total of 792 plots contained one invasive species, 427 plots contained two invasive species, and only nine plots contained three (S1 Table). As predicted, disturbance by humans increased the probability of invasion and the inclusion of land-use history significantly improved model fit (LR Χ^2^ = 28.50, df = 5, p < 0.001). However, the species count was significantly higher than in the always forested sites in only two land-use categories: agriculture to forest (log count = 0.28, z = 5.024, p < 0.001) and mined (log count = 0.23, z = 2.309, p = 0.021; Fig 2). Inclusion of distance to road did not improve model fit (LR Χ^2^ = 0.36, df = 1, p = 0.55).

### Invasive species abundance

**Fig 4:**
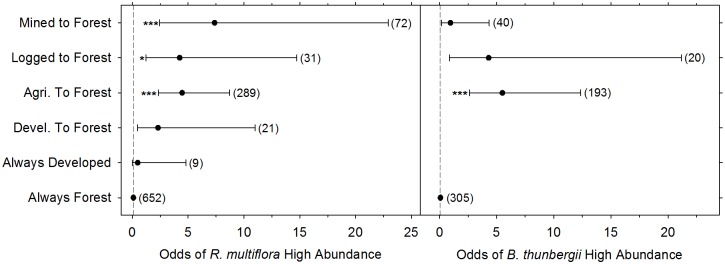
Odds of high abundance for (a) *R*. *multiflora*, (b) *B*. *thunbergii* with different land-use histories. Each point indicates the odds that each invasive species will be highly abundant (>10% cover) in a given land-use history ± 95% confidence intervals (*n* = 1074 plots for *R*. *multiflora* and *n* = 558 plots for *B*. *thunbergii*). Asterisks indicate a significant difference from always forested (i.e. forested continuously since at least 1939) plots (*p < 0.05, **p < 0.01, ***p < 0.001) and sample sizes are given in parentheses. Plots with *R*. *multiflora* or *B*. *thunbergii* cover class of 1–2 were categorized as low abundance plots and plots with cover classes of 3–6 were categorized as high abundance plots. Analysis of the always developed and developed to forest land-use categories were not complete for *B*. *thunbergii* due to small sample size.

When present, most invasive species were not abundant, with a median cover class of 2 (1–10% cover, classified as low invasive species abundance) for both *R*. *multiflora* and *B*. *thunbergii* (Fig 4). Most plots were in cover class 1 or 2 (70% of plots for *R*. *multiflora*, 76% for *B*. *thunbergii*), and very few had abundance over 50% cover (3.5% of plots for *R*. *multiflora*, 1.6% for *B*. *thunbergii*).

Abundance of *R*. *multiflora* varied by land-use history within PNR, with cover above ten percent (high abundance) in 19% of always forested plots, 47% of agriculture to forest plots, 29% of developed to forest plots, 35% of logged plots, 54% of mined plots, and 11% of developed plots. As predicted, disturbance by humans increased the probability that an invasive species would be highly abundant and the inclusion of land-use history significantly improved model fit (LR Χ^2^ = 28.18, df = 5, p < 0.001). The probability of high abundance of *R*. *multiflora* was significantly greater in all land-use categories compared to always forested sites, except sites that had experienced development (Fig 4a). For example, for a plot that was previously agricultural, the log odds of high *R*. *multiflora* abundance increased by 1.50 (Wald Z = 4.473, p < 0.001) and the odds increased by 4.46. For forest that had previously been logged, the log odds increased by 1.44 (Wald Z = 2.297, p = 0.022) and the odds by 4.24. Previously mined plots had the highest probability of having a high abundance of *R*. *multiflora* with the log odds increasing by 1.99 (Wald Z = 3.540, p = 0.0004) and the odds by 7.37. Proximity to roads did not significantly increase the probability of abundant *R*. *multiflora*, and inclusion of distance to road did not significantly improve model fit (LR Χ^2^ = 1.00, df = 1, p = 0.317).

Abundance of *B*. *thunbergii* varied by land-use history within PNR, with cover above ten percent in 13% of always forested plots, 43% of agriculture to forest plots, 30% of logged plots, and 12% of mined plots. *Berberis thunbergii* was not present at high abundance in the developed to forest plots or developed plots. As predicted, disturbance by humans increased the probability of high abundance and the inclusion of land-use history significantly improved model fit (LR Χ^2^ = 20.39, df = 5, p < 0.001). The probability of high abundance of *B*. *thunbergii* was significantly higher in agriculture to forest plots, where the log odds increased by 1.71 (Wald Z = 4.395, p < 0.001) and the odds by 5.50. There was no significant difference between always forested plots and previously mined plots (log odds = -0.05, Wald Z = -0.058, p = 0.95) or previously logged plots (log odds = 1.46, Wald Z = 1.827, p = 0.068). Plots with a history of development were not included in this model due to sample size limitations (n < 3). Furthermore, as with *R*. *multiflora*, proximity to roads did not significantly increase the probability of highly abundant *B*. *thunbergii*, and inclusion of distance to road did not significantly improve model fit (LR Χ^2^ = 1.08, df = 1, p = 0.298).

## Discussion

We found that a wide array of land-use histories significantly increased the likelihood of *R*. *multiflora* and *B*. *thunbergii* occurrence. Plots with a history of mining, logging, or agriculture were significantly more likely to have both invasive species than plots that had been continuously forested since 1939. *Rosa multiflora* was also more likely to occur in plots that were closer to roads and was more likely to occur in plots that had been developed than in plots that were always forested. By comparison, disturbance had less of an impact on the abundance of these invasive species after their introduction. Only a history of agriculture resulted in greater abundance of *B*. *thunbergii*, whereas *R*. *multiflora* was significantly more abundant in plots with mining, logging, or agricultural history. These results suggest that historic disturbances create “windows of opportunity” for introduction of invasive species, which results in long-term persistence of these species in the invaded ecosystem; however, effects of past disturbance on abundance following introduction are limited and depend on the type of disturbance.

### Mining

Our study is unique in examining the impacts of historic coal mining on the introduction and spread of invasive species along with other historic land-use changes. Strip mining for coal results in total vegetation clearing and a suite of changes to the soil [11], including compaction and the resultant decrease in water absorption [32], acidification, and loss of mycorrhizal symbionts [33], the seed bank, and soil microbes [10]. Furthermore, invasive species, such as *Elaeagnus umbellata*, are sometimes planted as part of the reclamation process because of their ability to spread quickly and grow under harsh conditions [10]. These alterations differ from modifications resulting from agriculture and other disturbances, but are similar in that they often inhibit re-establishment of native species while allowing disturbance specialist invasive species to establish. Our findings showed that mining significantly increased *R*. *multiflora* occurrence and abundance relative to plots that had always been forested. *Berberis*. *thunbergii* was also more likely to occur in areas that had historically been mined but, in contrast, was not more abundant where it did occur. Historically mined plots also contained more than twice the number of invasive plant species compared to plots that had been forested since 1939. As in our study, invasive species have been found in historically-mined areas, including *Latana camara* in coal fields in India [8] and autumn olive (*Elaeagnus umbellata*) and *R*. *multiflora* in historically-mined areas of Appalachia [9][31]. *Latana camara* and *E*. *umbellata* spread quickly in these areas after mining ceased, having negative effects on the recovery of native species, while spread of *R*. *multiflora* was not examined [8][9]. Our research implies that mine reclamation efforts should focus not only on soil amendments to decrease acidification and introduce mycorrhizal symbionts, but also on the establishment of native or non-invasive plant communities to limit invasion opportunities for species such as *R*. *multiflora* and *B*. *thunbergii*, which persist in the ecosystem for decades after mine abandonment.

### Agriculture

The increases in occurrence and abundance of *R*. *multiflora* and *B*. *thunbergii* in plots with a history of agriculture were similar in magnitude to the impacts of mining. Plots with a history of agriculture also contained significantly more invasive plant species than plots that had remained forested since 1939. *Berberis thunbergii* is a known post-agricultural specialist and a history of agriculture has been found to increase abundance and occurrence of many other invasive species including *R*. *multiflora*, *L*. *morrowii*, *A*. *petiolata*, and *C*. *orbiculata*, in addition to increasing the total number of invasive species found at a site [1][3][5] [34][35][36]. Agricultural abandonment creates an invasion opportunity, as with mining, by clearing native vegetation. As some invasive plant species were once planted in hedgerows or to prevent erosion, they are ideally located to take advantage of abandoned fields [37]. Further, agriculture often results in persistent changes in soil chemistry, as fertilizer application increases nitrogen and phosphorus concentrations for decades after the farmland is abandoned [38]. These highly fertile sites have been shown to increase growth of invasive species relative to forested areas [39]. Our results suggest that the disturbance and removal of the native plant community as a result of historic agriculture allowed for the initial invasion of *R*. *multiflora* and *B*. *thunbergii*, and thus resulted in a greater probability of invasive occurrence even after the forest regrew in those plots. However, historic agriculture is unique among our land-use history categories in that it increased the abundance of both invasive focal species as well. These results suggest that a history of agriculture continues to enhance invasive species performance for decades after abandonment perhaps due to long-term increases in soil fertility. Further, our findings highlight the importance of re-establishing a native plant community quickly after farming is discontinued to limit invasive species introduction and spread.

### Other land-use histories

Past logging increased the occurrence of both invasive species at PNR. It also increased the probability of high abundance of *R*. *multiflora* compared with plots that had always been forested. The effects of logging on invasion dynamics vary significantly among studies, with some finding results similar to ours for *B*. *thunbergii* [40], while others suggest logging is associated with both increased occurrence and abundance of invasive species, as we saw in *R*. *multiflora* [41]. This variability among studies may be due to differing intensity of logging among studies and interactions with soil fertility characteristics. Low intensity logging typically results in limited change in species composition and is primarily associated with release of shade-tolerant, late successional native species [34]. In contrast, high intensity logging may result in increased opportunities for invasion by non-native species, particularly when combined with high soil nitrogen levels. Occurrence of both *R*. *multiflora* and *B*. *thunbergii*, along with several other invasive species was associated with high intensity logging, but only if the carbon to nitrogen ratio in the soil was relatively low [42].

Historic and present development increased the likelihood of *R*. *multiflora* occurrence, whereas development had no impacts on *B*. *thunbergii* occurrence or abundance. In one of the few studies that examined the effects of continuous development on *R*. *multiflora* and *B*. *thunbergii*, Mosher et al. [5] found that both species were significantly more abundant in areas that were continuously developed than in areas that stayed forested, in contrast to our results; however they did not have a category for areas that had been developed and then allowed to return to forest. Another study found that high levels of present development corresponded with higher frequency and cover of invasive species and that this pattern held true to a certain extent for both *R*. *multiflora* and *B*. *thunbergii* [2]. However, that study had only three sites and compared the effects of past and present land use, unlike our study, which created land-use categories based on both past and present land use. Most of the developed plots at PNR are of relatively low disturbance intensity (i.e., lawns). This low level of disturbance may have resulted in the limited effects of development on invasive species occurrence and abundance. Studies of the impacts of previous development followed by reforestation on non-native species invasion are quite rare as the conversion of forest to developed land is much more common than the reverse [6]. Thus, our study is unique in contributing to our limited understanding of both the temporal sequence of land-use change and the effects of present and historic development.

### Roads

We found that *R*. *multiflora* was more likely to occur in plots closer to a road, even when land-use history was accounted for in the model. However, there was no difference in the probability of high abundance of *R*. *multiflora* with differing proximity to roads and the occurrence and abundance of *B*. *thunbergii* were not affected by distance to roads. Roads are important in the spread of invasive species as they provide corridors for their spread. Roads also provide suitable habitat for invasive species as the disturbance created by roads often leads to fewer competitors and increased nutrient, moisture, and light availability [12][13][14]. Additionally, roads are hypothesized to increase propagule pressure of invasive species thereby increasing the success of invasion into surrounding forest [4]. The effects of roads on the occurrence and abundance of invasive species tend to vary among studies depending on which species are examined and which other variables are included in the model. For instance, Flory and Clay [13] examined the impacts of proximity to roads on abundance of seven invasive species. Four of the seven species studied, including *B*. *thunbergii*, increased in abundance closer to roads, whereas the abundance of *R*. *multiflora* and two others was not significantly related to distance to roads. However, that study did not include land-use in its models. Models such as ours that include both land-use history and proximity to roads as drivers of invasive species occurrence and abundance are essential. Simultaneously testing the effects of land-use and road proximity allows the effects of each to be assessed independently as our models include variation accounted for by all other terms when assessing the significance of each variable in the model. Thus, factors like external propagule pressures associated with proximity to roads do not confound our estimates of invasive species occurrence and abundance in plots with differing land-use histories. Of the three studies that have included land-use history in their models, one found no effect of proximity to roads on invasive species occurrence or abundance [15], whereas the other two found that proximity to roads affected abundance, but not occurrence of invasive species, opposite to our findings [2][16]. However, all three of these analyses pooled results from multiple invasive species, with one study using data from 95 species, so effects on individual species may have been masked in these analyses. Given that our findings and those of Flory and Clay [13] both showed species-specific effects of proximity to roads, we suggest that individual analyses of invasive species are most appropriate for assessment of disturbance and land-use impacts on occurrence and abundance.

## Conclusions

This study highlights the importance of historic land-use for informing predictions of invasion. A history of mining, agriculture, logging, development, and proximity to roads all increased the probability that at least one of the invasive species that we studied would occur in a particular plot. In contrast, fewer land uses were associated with increased probability of high abundance in *R*. *multiflora* and *B*. *thunbergii* or with higher total number of invasive plant species, though the most disturbing land uses of mining and agriculture were associated with these variables. We are not the first study to note that invasive species abundance may be driven by different environmental and anthropogenic factors than occurrence of these species [1]. However, since the abundance, rather than occurrence, of invasive species is related to decreases in community diversity [43], our research suggests it may be important to focus our invasive species eradication efforts in areas with previous agriculture or mining.

## Supporting Information

S1 Figs Analysis of Spatial Autocorrelation. **Figure A. Plot of residual semivariance vs. distance (m) in *R*. *multiflora* occurrence models without (a) and with (b) Block as a random effect**. Distance refers to the distance between the plots from which a given pair of data points were collected. Dashed lines indicate 95% confidence intervals (CI) and the *p*-value given is for Moran’s I test of spatial autocorrelation. Inclusion of Block in the *R*. *multiflora* occurrence models greatly reduced spatial autocorrelation between plots in different blocks although some spatial autocorrelation remains between plots within the same Block (*p* < 0.05). **Figure B. Plot of residual semivariance vs. distance (m) in *R*. *multiflora* abundance models without (a) and with (b) Block as a random effect**. Distance indicates the distance between the plots from which a given pair of data points were collected. Dashed lines give 95% confidence intervals (CI) and the *p*-value is for Moran’s I test of spatial autocorrelation. Inclusion of Block in the *R*. *multiflora* abundance models eliminated spatial autocorrelation between plots at least 50 meters apart although some spatial autocorrelation remains between plots within the same Block (*p* < 0.05). **Figure C. Plot of residual semivariance vs. distance (m) in *B thunbergii* occurrence models without (a) and with (b) Block as a random effect**. Distance provides the distance between the plots from which a given pair of data points were collected. The dashed lines give 95% confidence intervals (CI) and the *p*-value given is for Moran’s I test of spatial autocorrelation. Inclusion of Block in the *B*. *thunbergii* occurrence models eliminated all spatial autocorrelation in the model residuals (*p* > 0.05). **Figure D. Plot of residual semivariance vs. distance (m) in *B*. *thunbergii* abundance models without (a) and with (b) Block as a random effect**. Distance indicates the distance between the plots from which a given pair of data points were collected. The dashed lines give 95% confidence intervals (CI) and the *p*-value given is for Moran’s I test of spatial autocorrelation. Inclusion of Block in the *B*. *thunbergii* abundance models eliminated all spatial autocorrelation in the model residuals (*p* > 0.05).
(DOCX)

S1 Table Full Dataset. **This table provides plot and block level information for every plot and block included in this study**. Among others the table includes plot and block identification, distances to a road, and occurrence and cover class (abundance) for both *R*. *multiflora* and *B*. *thunbergii*.
(XLSX)
