# Supplementary material for: Historic Mining and Agriculture as Indicators of Occurrence and Abundance of Widespread Invasive Plant Species
Source: PLoS One. 2015 Jun 5;10(6):e0128161. doi: 10.1371/journal.pone.0128161 (PMC4457889; doi:10.1371/journal.pone.0128161)
Supplement: S1 Figs — Figure A. Plot of residual semivariance vs. distance (m) in R. multiflora occurrence models without (a) and with (b) Block as a random effect. Distance refers to the distance between the plots from which a given pair of data points were collected. Dashed lines indicate 95% confidence intervals (CI) and the p-value given is for Moran’s I test of spatial autocorrelation. Inclusion of Block in the R. multiflora occurrence models greatly reduced spatial autocorrelation between plots in different blocks although some spatial autocorrelation remains between plots within the same Block (p < 0.05). Figure B. Plot of residual semivariance vs. distance (m) in R. multiflora abundance models without (a) and with (b) Block as a random effect. Distance indicates the distance between the plots from which a given pair of data points were collected. Dashed lines give 95% confidence intervals (CI) and the p-value is for Moran’s I test of spatial autocorrelation. Inclusion of Block in the R. multiflora abundance models eliminated spatial autocorrelation between plots at least 50 meters apart although some spatial autocorrelation remains between plots within the same Block (p < 0.05). Figure C. Plot of residual semivariance vs. distance (m) in B thunbergii occurrence models without (a) and with (b) Block as a random effect. Distance provides the distance between the plots from which a given pair of data points were collected. The dashed lines give 95% confidence intervals (CI) and the p-value given is for Moran’s I test of spatial autocorrelation. Inclusion of Block in the B. thunbergii occurrence models eliminated all spatial autocorrelation in the model residuals (p > 0.05). Figure D. Plot of residual semivariance vs. distance (m) in B. thunbergii abundance models without (a) and with (b) Block as a random effect. Distance indicates the distance between the plots from which a given pair of data points were collected. The dashed lines give 95% confidence intervals (CI) and the p-value [file pone.0128161.s001.docx]

**S1 Figs. Analysis of Spatial Autocorrelation.**


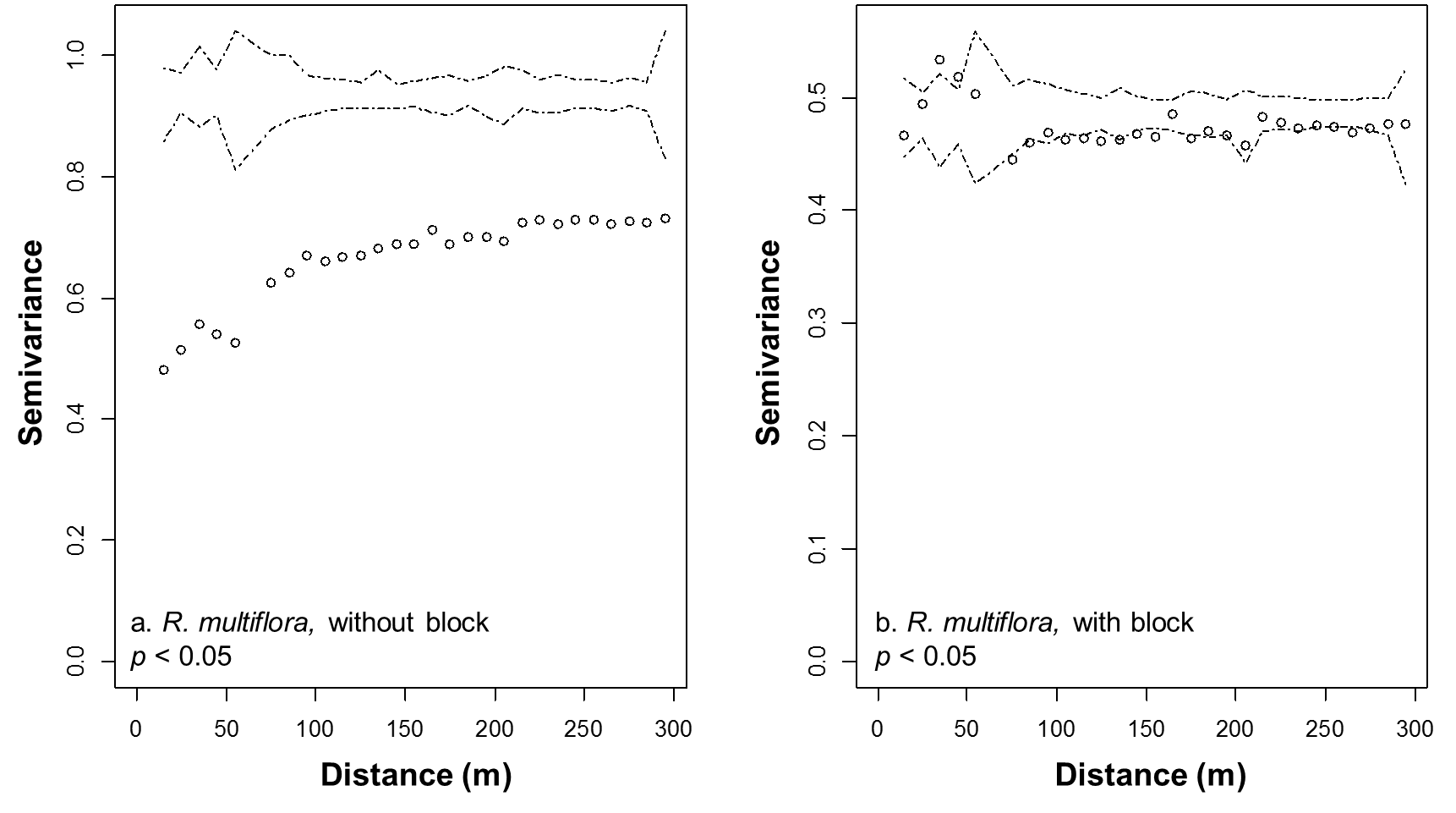


**Figure A in S1 Figs**. Plot of residual semivariance vs. distance (m) in *R. multiflora* occurrence models without (a) and with (b) Block as a random effect. Distance refers to the distance between the plots from which a given pair of data points were collected. Dashed lines indicate 95% confidence intervals (CI) and the *p*-value given is for Moran’s I test of spatial autocorrelation. Inclusion of Block in the *R. multiflora* occurrence models greatly reduced spatial autocorrelation between plots in different blocks although some spatial autocorrelation remains between plots within the same Block (*p* < 0.05).


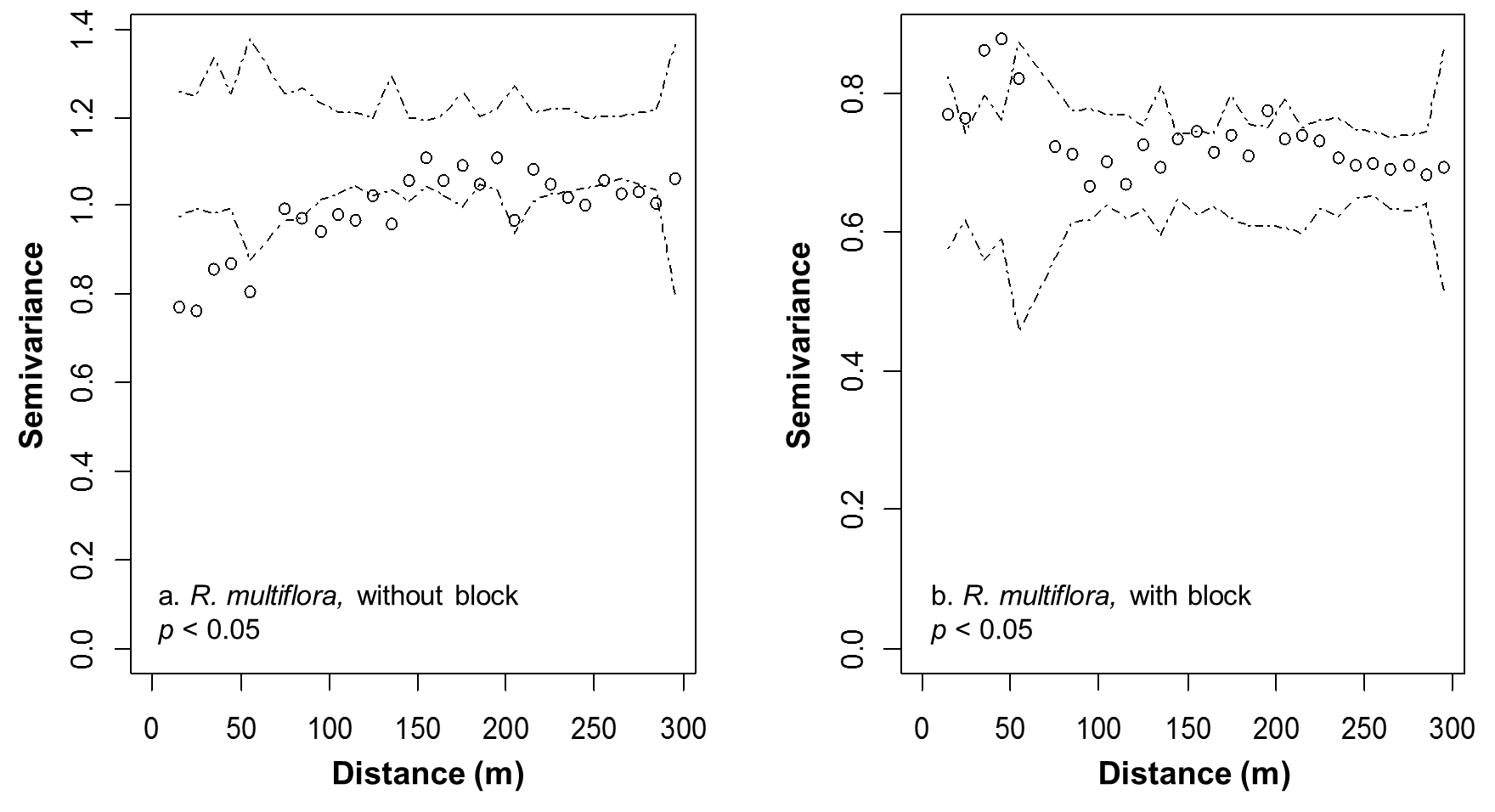


**Figure B in S1 Figs**. Plot of residual semivariance vs. distance (m) in *R. multiflora* abundance models without (a) and with (b) Block as a random effect. Distance indicates the distance between the plots from which a given pair of data points were collected. Dashed lines give 95% confidence intervals (CI) and the *p*-value is for Moran’s I test of spatial autocorrelation. Inclusion of Block in the *R. multiflora* abundance models eliminated spatial autocorrelation between plots at least 50 meters apart although some spatial autocorrelation remains between plots within the same Block (*p* < 0.05).


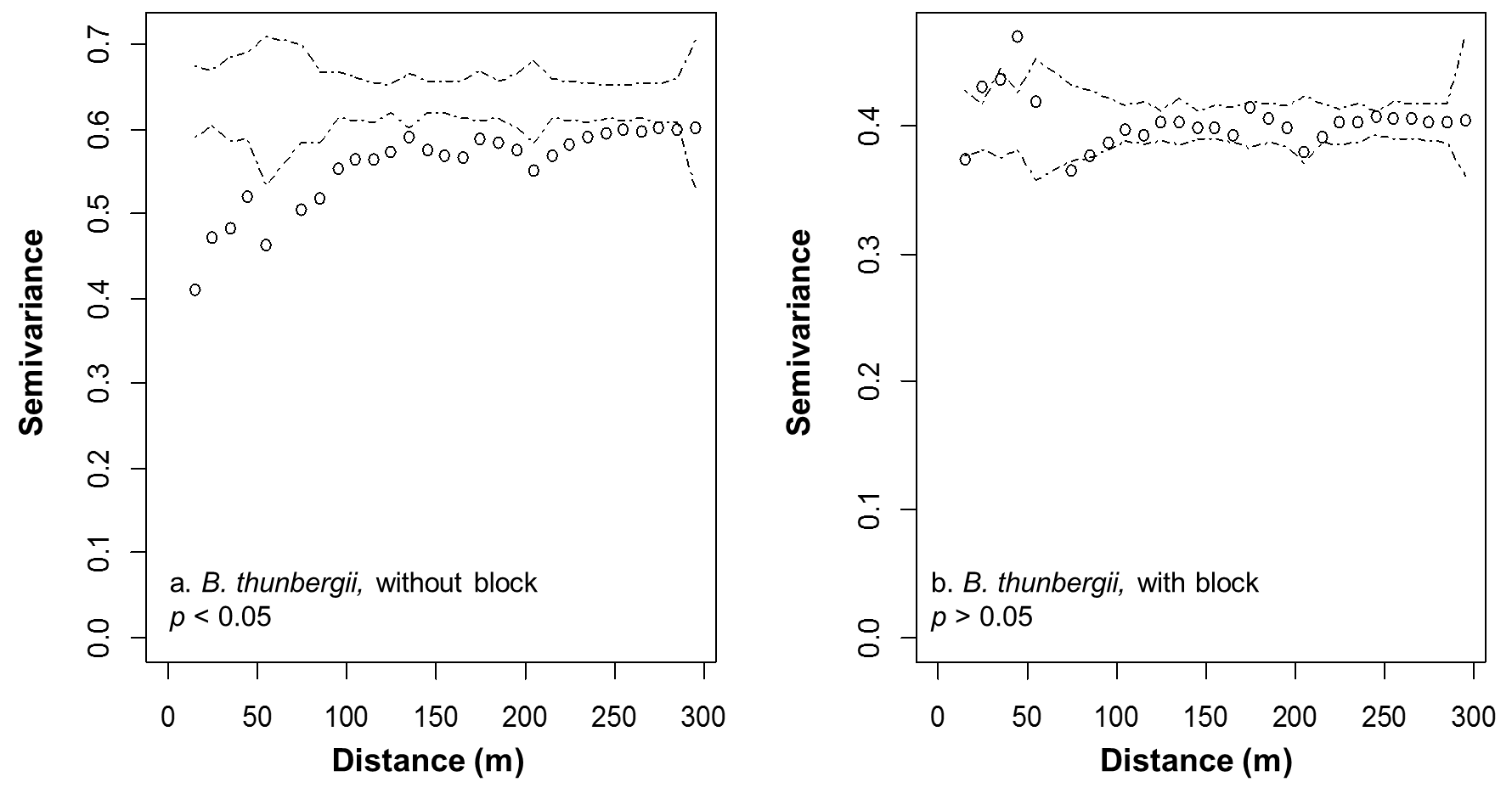


**Figure C in S1 Figs**. Plot of residual semivariance vs. distance (m) in *B. thunbergii* occurrence models without (a) and with (b) Block as a random effect. Distance provides the distance between the plots from which a given pair of data points were collected. The dashed lines give 95% confidence intervals (CI) and the *p*-value given is for Moran’s I test of spatial autocorrelation. Inclusion of Block in the *B. thunbergii* occurrence models eliminated all spatial autocorrelation in the model residuals (*p* > 0.05).


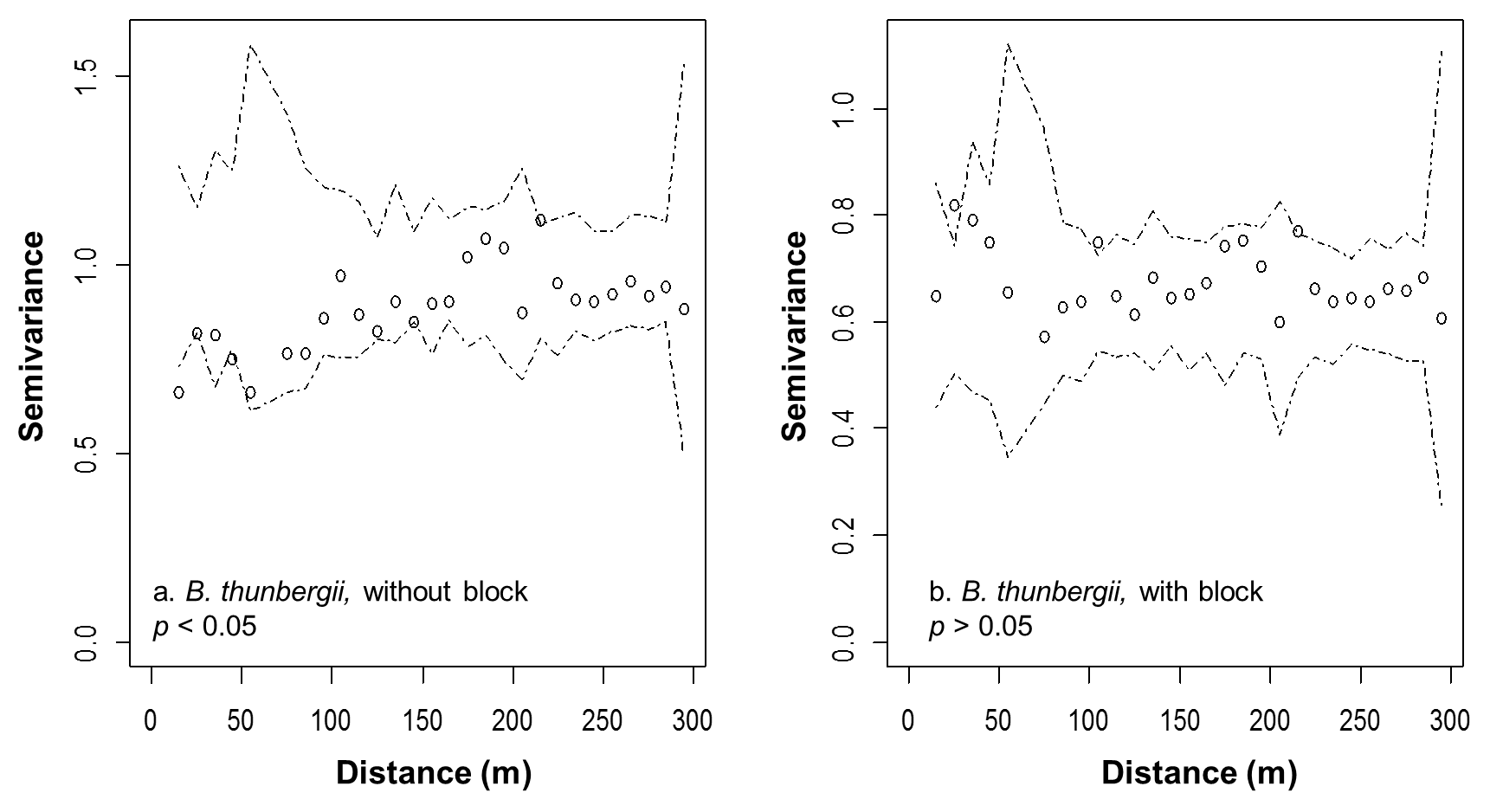


**Figure D in S1 Figs**. Plot of residual semivariance vs. distance (m) in *B. thunbergii* abundance models without (a) and with (b) Block as a random effect. Distance indicates the distance between the plots from which a given pair of data points were collected. The dashed lines give 95% confidence intervals (CI) and the *p*-value given is for Moran’s I test of spatial autocorrelation. Inclusion of Block in the *B. thunbergii* abundance models eliminated all spatial autocorrelation in the model residuals (*p* > 0.05).
